# Oral vaccination with a recombinant *Salmonella *vaccine vector provokes systemic HIV-1 subtype C Gag-specific CD4+ Th1 and Th2 cell immune responses in mice

**DOI:** 10.1186/1743-422X-6-87

**Published:** 2009-06-25

**Authors:** Nyasha Chin'ombe, William R Bourn, Anna-Lise Williamson, Enid G Shephard

**Affiliations:** 1 Institute of Infectious Disease and Molecular Medicine, Faculty of Health Sciences, University of Cape Town, Observatory 7925, Cape Town, South Africa; 2 National Health Laboratory Services, Groote Schuur Hospital, Cape Town, South Africa; 3 Department of Medicine, Faculty of Health Sciences, University of Cape Town, Observatory 7925, Cape Town, South Africa; 4 Kapa Biosystems (Pty) Ltd, Observatory 7925, Cape Town, South Africa

## Abstract

**Background:**

Recombinant *Salmonella *vaccine vectors may potentially be used to induce specific CD4+ T cell responses against foreign viral antigens. Such immune responses are required features of vaccines against pathogens such as human immunodeficiency virus type 1 (HIV-1). The aim of this study was to investigate the induction of systemic HIV-1-specific CD4+ T helper (Th) responses in mice after oral immunization with a live attenuated *Salmonella *vaccine vector that expressed HIV-1 subtype C Gag. Groups of BALB/c mice were vaccinated orally three times (4 weeks apart) with this recombinant *Salmonella*. At sacrifice, 28 days after the last immunization, systemic CD4+ Th1 and Th2 cytokine responses were evaluated by enzyme-linked immunospot assay and cytometric bead array. HIV-1 Gag-specific IgG1 and IgG2a humoral responses in the serum were determined by enzyme-linked immunosorbent assay.

**Results:**

Mice vaccinated with the recombinant *Salmonella *elicited both HIV-1-specific Th1 (interferon-gamma (IFN-γ) and tumour necrosis factor-alpha (TNF-α)) and Th2 (interleukin-4 (IL-4) and interleukin-5 (IL-5)) cytokine responses. The vaccine induced 70 (IFN-γ) spot-forming units (SFUs)/10e6 splenocytes and 238 IL-4 SFUs/10e6 splenocytes. Splenocytes from vaccinated mice also produced high levels of Th1 and Th2 cytokines upon stimulation with a Gag CD4 peptide. The levels of IFN-γ, TNF-α, IL-4 and IL-5 were 7.5-, 29.1-, 26.2- and 89.3-fold above the background, respectively. Both HIV-1 Gag-specific IgG1 and IgG2a antibodies were detected in the sera of vaccinated mice.

**Conclusion:**

The study highlights the potential of orally-delivered attenuated *Salmonella *as mucosal vaccine vectors for HIV-1 Subtype C Gag to induce Gag-specific CD4+ Th1 and Th2 cellular immune responses and antibodies which may be important characteristics required for protection against HIV-1 infection.

## Background

Attenuated *Salmonella *bacterial vaccines may be exploited for use as vectors for the oral delivery of HIV-1 antigens to both the mucosal and systemic compartments of the immune system. The bacteria provoke potent mucosal and systemic immune responses when administered by the oral route [1-4]. After oral administration, the bacteria are taken-up by professional phagocytes in the gut; they can then spread throughout the intestinal lymphatic tissues and reach the systemic compartments such as the liver and the spleen. In the phagocytes, the bacteria are found in *Salmonella*-containing vacuoles, or phagosomes, and the antigens are predominantly targeted to the MHC Class II presentation pathway, thereby provoking mainly the CD4+ Th1 and Th2 responses [5]. The induction of antigen-specific CD4 Th1 and Th2 responses is important for protection against infection by various types of pathogens. CD4+ Th1 cells produce cytokines such as IFN-γ, IL-2 and TNF-α, while CD4+ Th2 cells produce cytokines such as IL-4 and IL-5 [6-8]. In the case of viral infection, CD4+ Th1 and Th2 cells play a critical role in maintaining CD8+ T cell and antibody responses, respectively [9,10]. These cells are, therefore also indirectly important in their control of viral replication and vireamia [11]. In the present study, we investigated the induction of systemic antigen-specific CD4+ Th1 and Th2 cell responses in mice that had been orally vaccinated with a recombinant *Salmonella enterica *serovar Typhimurium *aro*C vaccine vector that expressed codon-optimized HIV-1 subtype C Gag antigen.

## Methods

### Bacterial strains and culture conditions

*Escherichia coli *SCS110 cells (Stratagene, USA) were used for genetic manipulations and cloning. The *Salmonella enterica *serovar Typhimurium Δ*aro*C mutant vaccine strain (TML-MD58) was supplied by Microscience Pty Ltd (United Kingdom). This mutant has a deletion in the *aro*C gene, which encodes chorismate synthase, an enzyme that is crucial for the biosynthesis of tryptophan, tyrosine, phenylalanine, para-aminobenzoic acid and 2,3-dihydroxybenzoate [12]. 2YT media (supplemented, where necessary, with ampicillin and the aromatic amino acids) was used for culture of the recombinant *Salmonella*.

### Construction of a Gag expression cassette

To construct the *Salmonella *Gag-expression plasmid, a codon-optimized HIV-1 *gag *gene, synthesized for us by Geneart (USA), was cloned by standard recombinant DNA protocols [13] into pGEM+GFP, a plasmid designed to express green fluorescent protein that we previously constructed [14]. The *gfp *gene in pGEM+GFP was replaced with the *gag *gene and the plasmid designated pGEM+Gag was generated. The expression of Gag was under the control of the *E. coli lac *(lactose) promoter. Competent Δ*aro*C *Salmonella enterica *serovar Typhimurium mutant was transformed with the expression plasmid (pGEM+Gag) and this resulted in the generation of a recombinant *Salmonella *vaccine clone, designated, aroC+Gag. The parent cloning vector, pGEM^®^-T Easy (Promega), was used as a negative control plasmid to generate a vaccine designated aroC+pGEM. The expression of HIV-1 Gag in the recombinant *Salmonella *bacteria, aroC+Gag, was assessed by SDS-PAGE according to standard protocols. The expression of the Gag protein was further confirmed using the Roche Elecsys^® ^HIV p24 Ag assay (Roche) according to manufacturer's recommendations.

### *Salmonella *vaccine stocks

Stocks of recombinant *Salmonella *bacterial vaccines were prepared from culture colonies of AroC+Gag (test vaccine) or aroC+pGEM (negative control vaccine). A single colony was inoculated into 200 ml of 2YT liquid media supplemented with ampicillin (100 μg/ml) and aromatic amino acids and grown at 37°C with vigorous aeration. The bacterial cells were harvested when they reached logarithmic phase (OD_600 _of 0.8) by centrifugation and washed once with PBS (pH 7.4). The vaccine was suspended in PBS with 15% glycerol and stored at -80°C prior to use for immunization.

### Immunization of mice and preparation of splenocytes

Animal work was approved by the University of Cape Town Animal Ethics Committee. Female H-2^d ^BALB/c mice (8–10 weeks old) were purchased from South African Vaccine Producers (Johannesburg, South Africa) and were housed (5 per group) at the University of Cape Town Animal Unit. The mice were allowed to adapt to the new environment for at least 10 days before immunization. They were bled on day 0 prior to being inoculated by intragastric gavage with 1 × 10e8 colony forming units (CFUs)/mouse of either the *Salmonella *test vaccine (aroC+Gag) or the negative control (aroC+pGEM) and again on days 28 and 56 prior to the booster *Salmonella *vaccine being given. Blood was also taken on day 84 just prior to sacrifice of the mice for harvest of spleens. Serum was collected and used in ELISA assays to measure Gag-specific antibody responses.

Spleens were harvested from the mice at sacrifice on day 84. A pool of 5 spleens per group was made, then passed through a 70-μm cell strainer to obtain a single cell suspension of splenocytes. Splenocytes were suspended in R10 medium (RPMI-1640 with 10% heat inactivated fetal calf serum, 15 mM β-mercaptoethanol, 100 U penicillin and 100 μg streptomycin per ml). Red cells were lysed using erythrocyte lysing solution (0.15 M NH_4_Cl, 10 mM KHCO_3_, 0.1 mM Na_2_EDTA). The splenocytes were suspended at a concentration of 5 × 10e6 cells per ml for use in enzyme-linked immunospot (ELISPOT) assays or at a concentration of 15 × 10e6 cells per ml for stimulation with Gag peptides to quantify Gag-specific cytokines released into the supernatant.

### IFN-γ and IL-4 ELISPOT assays

Gag-specific CD4+ T cells secreting IFN-γ or IL-4 in the spleen were enumerated using IFN-γ and IL-4 ELISPOT assay kits (BD Pharmingen) as previously described [15]. Briefly, splenocytes were cultured in triplicate wells at 0.5 × 10e6 cells/well in a final volume of 200 μl with either R10 medium only (background responses) or R10 medium containing the Gag CD4 peptide (NPPIPVGDIYKRWIILGLNK, an MHC class II-restricted, CD4 binding peptide) at 4 μg/ml. After incubation (37°C, 5% CO_2_) for 24 hours (IFN-γ ELISPOT assay) or 48 hours (IL-4 ELISPOT assay), the cells secreting IFN-γ or IL-4 were detected using Nova Red substrate (Vector Labs) according to the manufacturer's instructions. Spots were counted using CTL Analyzer and ImmunoSpot Version 3.2 software (Cellular Technology, USA). The mean number of spots ± SD in triplicate wells was calculated and expressed as spot-forming units (SFU) per 10e6 splenocytes. Differences in immune responses between vaccine groups were analyzed by a two-sample student's t-test. A p < 0.05 was considered statistically significant. The differences in response between stimulated cells and unstimulated cells within the same vaccine group were also analyzed by a student's t-test and p < 0.05 was considered statistically significant.

### Quantification of Gag-specific CD4+ Th1 and Th2 cytokine production

Splenocytes (1.5 × 10e6) were cultured (37°C, 5% CO_2_) in a final volume of 200 μl R10 medium only (background responses) or R10 medium containing the Gag CD4 peptide (as used in the ELISPOT assays) at 4 μg/ml [15]. Culture supernatants were harvested at 48 hours and the content of Gag-specific Th1 cytokines (IFN-γ and TNF-α) and Th2 cytokines (IL-4 and IL-5) were quantified using a mouse Th1/Th2 Cytokine Bead Array (CBA) assay (BD Pharmingen) according to manufacturer's instructions. Results were expressed as pg cytokine per 10e6 splenocytes. Differences in immune responses between vaccine groups were analyzed by a two-sample student's t-test and p < 0.05 was considered statistically significant. The differences in response between stimulated cells and unstimulated cells within the same group were also analyzed by a student's t-test and p < 0.05 was considered statistically significant.

### HIV-1 Gag antibody assay

Antibodies (total IgG) to HIV Gag were detected in mouse serum (pooled from 5 mice per group) collected on day 0, 28, 56 and 84 and Gag-specific IgG1 and IgG2a detected at day 84 using HIV-1 Gag (P55) enzyme linked immunosorbent assay (ELISA). Briefly, flat-bottom, 96-well MaxiSorp ELISA plates (AEC-Amersham) were coated with 50 μg of HIV-1 P55 (Quality Biological Inc. USA) in 50 μl 0.1 M bicarbonate buffer (pH 9.5) overnight at 4°C. Plates were washed with PBS-Tween (PBS containing 0.3% Tween 20, Merck) and blocked with blocking solution (PBS containing 0.3% Tween 20, 1% goat serum, 3% milk powder) overnight at 4°C. Mouse serum samples were diluted (1/100), and 100 μl added to duplicate wells and the reaction incubated overnight at 4°C. After washing with PBS-Tween containing 1% goat serum and 1% milk powder, total Gag (P55)-specific IgG, was detected by the addition of 100 μl/well of biotinylated goat anti-mouse IgG (Southern Biotechnology, Birmingham, AL). After incubation at 37°C for 2 hours, plates were washed with PBS-Tween and streptavidin-alkaline phosphatase (50 μl/well) was added and the plates incubated a further 1 hour at 37°C. To detect HIV-1 Gag (P55)-specific IgG1 and IgG2a isotypes, alkaline phosphatase-conjugated antibodies specific for IgG1 or IgG2a) (Serotec) were used. Alkaline phosphatase was reacted with 50 μl/well para-nitrophenyl phosphate (PNP) diluted to a final concentration of 1 mg/ml. The optical density (OD) was read at 405 nm and means of duplicates was calculated. For total IgG, the ratios of vaccinated to prebleed were calculated and represented graphically. The OD readings were represented graphically for IgG1 and IgG2a. Differences in immune responses between vaccine groups were analyzed by a two-sample student's t-test and a p value < 0.05 was considered statistically significant.

## Results

### Construction of a codon-optimized HIV-1 Gag-expressing *Salmonella *vaccine vector

**Figure 1:**
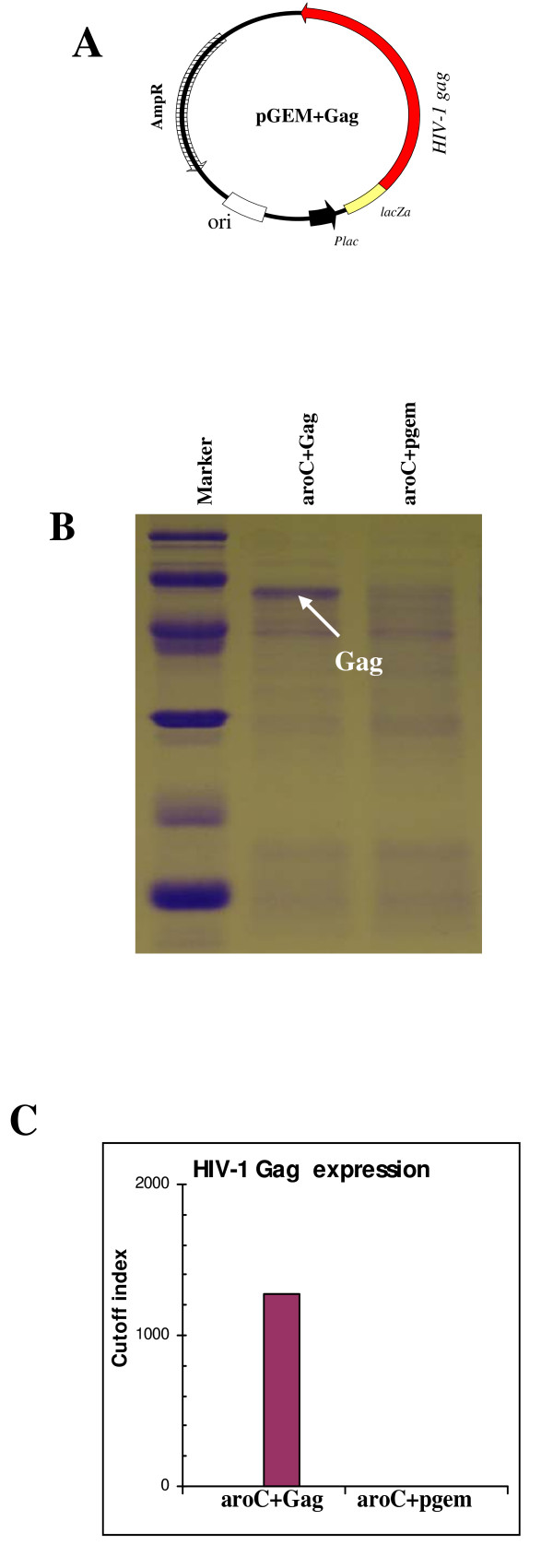
**The HIV-1 subtype C Gag expression plasmid (pGEM+Gag) and Gag expression by *Salmonella***. (A) The HIV Gag expression cassette contained the *gag *gene fused in-frame with the β-galactosidase α-gene and expression was under the *E. coli lac *(*lactose*) promoter. The plasmid contained an *E. coli *origin of replication (ori) and ampicillin resistance gene (AmpR). (B) The relative expression of the Gag by the recombinant *Salmonella *vaccine (aroC+Gag) was determined by SDS-PAGE and (C) the Roche Elecsys^® ^HIV p24 Ag assay. In the Roche Elecsys^® ^HIV p24 Ag assay, total bacterial protein lysate was diluted 1/1000 in water and the cut-off index was calculated by the Elecsys^® ^2010 analyzer using readings from the negative and positive calibrators.

A prokaryotic expression plasmid that contained a wild-type HIV-1 Subtype C *gag *gene was initially constructed. As Gag expression was poor, probably because of the presence of bacterial rare codons (data not shown), we redesigned the recombinant vaccine. For this the wild-type HIV-1 *gag *gene was replaced with a synthesised gene that reflected codon usage in *Salmonella *and the expression plasmid, pGEM+Gag, containing the codon-optimized *gag *was successfully constructed (Figure 1A). The plasmid contained the HIV-1 *gag *gene fused to the β-galactosidase α-fragment and the expression was constitutively driven by the *E. coli lac *promoter and other *lac *operon transcription and translation domains found in pGEM-Teasy plasmid. The recombinant plasmid-carrying *Salmonella *expressed very high levels of HIV Gag (Figure 1B). The Gag protein band was visible in Coomassie blue-stained SDS-PAGE gels (Figure 1B, Lane 2). The high-level of Gag protein expressed by the *Salmonella *was further confirmed by Roche Elecsys^® ^HIV p24 Ag Capture assay (Figure 1C). The expression of codon-optimized Gag was higher than previously found with wild-type Gag (data not shown).

### Oral vaccination of mice with a recombinant *Salmonella *induces Th1 and Th2 cytokine producing CD4+ T cells

**Figure 2:**
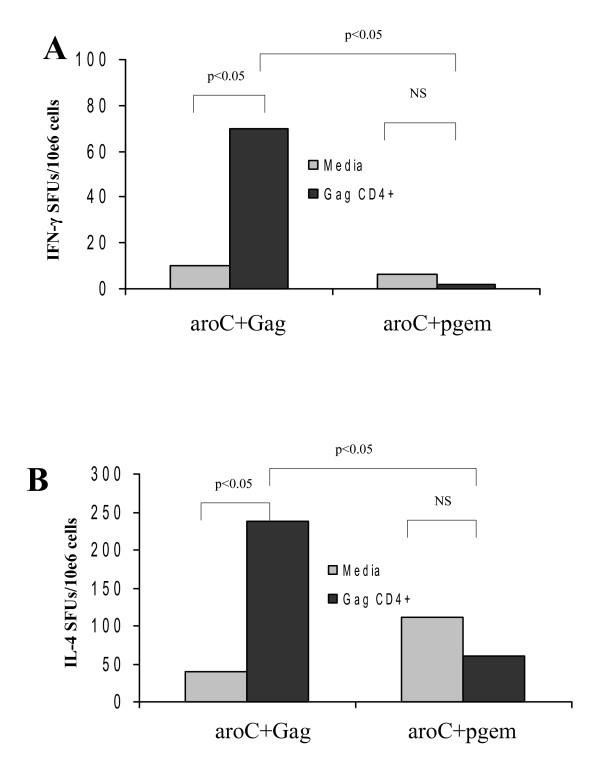
**HIV-1 subtype C Gag-specific CD4+ Th1 and Th2 cell responses as evaluated by IFN-γ and IL-4 ELISPOT assays**. Groups of mice (5 mice per group) were vaccinated three times (day 0, 28 and 56) with live recombinant *Salmonella *vaccine that expressed HIV-1 Subtype C Gag (aroC+Gag) or an antigen-negative *Salmonella *control vaccine (aroC+pGEM). Mice were sacrificed on day 84, and splenocytes were prepared from isolated spleens pooled from 5 mice per group then used in IFN-γ (A) and IL-4 (B) ELISPOT assays with R10 medium only (negative assay control) or with the Gag CD4 peptide. Bars are the mean number of spots from triplicate wells and are expressed as SFU/10e6 splenocytes. Differences in immune responses between vaccine groups were analyzed by a two-sample student's t-test. A p < 0.05 was considered statistically significant. The differences in response between stimulated cells and unstimulated cells within the same vaccine group were also analyzed by a student's t-test and a p < 0.05 was considered statistically significant. A p > 0.05 was considered not statistically significant (NS).

CD4+ Th1/Th2 T cells induced in the spleens of mice in response to oral vaccination with the recombinant codon-optimized HIV-1 Gag-expressing *Salmonella *vaccine vector, aroC+Gag, were evaluated using ELISPOT assays. Mice vaccinated with aroC+Gag developed HIV-1 Gag-specific CD4+ Th1 (IFN-γ) and Th2 (IL-4) cells in the spleen. The frequency of Gag-specific IFN-γ spot-forming units from aroC+Gag was above the background (p < 0.05) (Figure 2A). Cells from the aroC+Gag group responded to Gag CD4 peptide stimulation. The number of IL-4-producing cells from aroC+Gag was also significantly higher than the number of cells producing IL-4 from the negative vaccine control (p < 0.05) (Figure 2B). It was, therefore, evident that both Gag-specific IFN-γ and IL-4 cytokines were elicited by the recombinant *Salmonella *vaccine vector.

**Figure 3:**
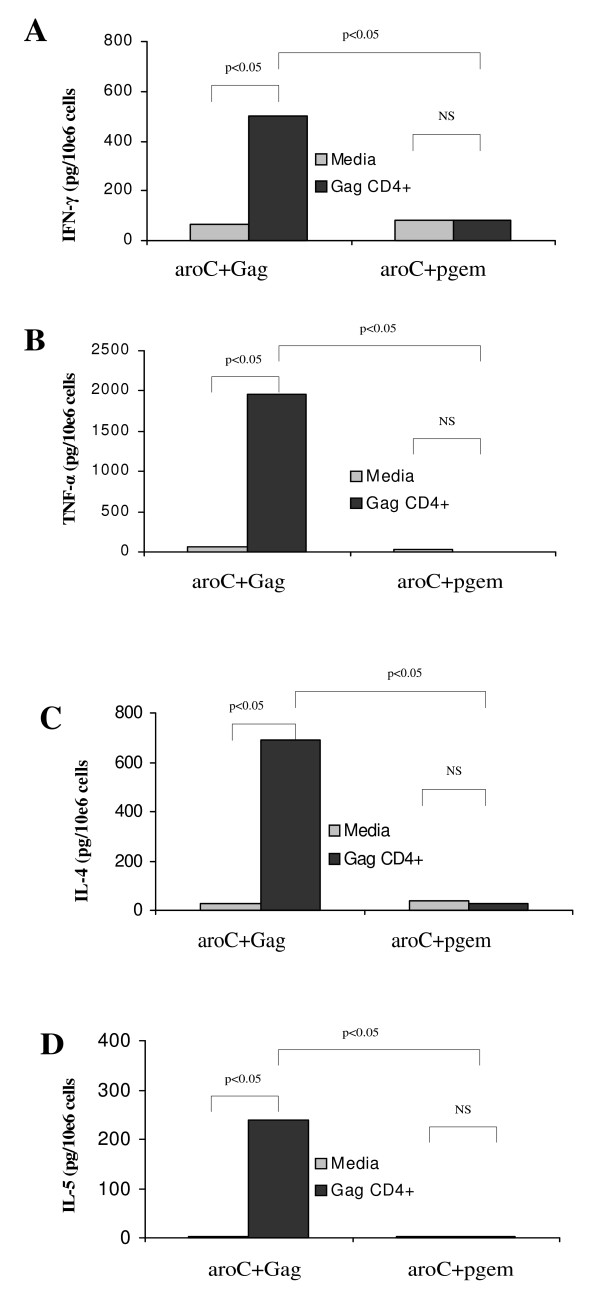
**HIV-1 subtype C Gag-specific CD4+ Th1 and Th2 cell responses as evaluated by the amount of cytokines secreted by stimulated cells**. Groups of mice (5 per group) were vaccinated with live recombinant *Salmonella *vaccine that expressed HIV-1 Subtype C Gag (aroC+Gag) or antigen-negative *Salmonella *control vaccine (aroC+pGEM) as indicated in Figure 2. Splenocytes isolated and pooled from 5 mice per group on day 84 were incubated in R10 medium only (negative assay control), or stimulated with a Gag CD4 peptide for 48 hrs. Cytokines released into the supernatant were quantified using a mouse Th1/Th2 cytokine bead array (CBA) assay. (A) IFN-γ, (B) TNF-α, (C) IL-4 and (D) IL-5. Each bar in the graphs represents the average picogram amount of cytokine produced per 10e6 splenocytes in 48 hours of stimulation with media or Gag CD4 peptide, for triplicate responses. A p < 0.05 was considered statistically significant. A p > 0.05 was considered not statistically significant (NS).

A CBA assay was used to quantify Gag-specific IFN-γ, TNF-α, IL-4 and IL-5 cytokines secreted by the splenocytes upon antigenic stimulation. The amounts of CD4+ Th1 (INF-γ and TNF-α) and Th2 (IL-4 and IL-5) cytokines secreted by the splenocytes into the supernatant were quantified. The levels of Gag-specific Th1 and Th2 cytokines were all significantly elevated above the background (p < 0.05) (Figure 3) in mice vaccinated with aroC+Gag. The levels of Th1 cytokines, IFN-γ and TNF-α were 7.5-fold and 29.1-fold above the background, respectively (Figure 3A and 3B). The Th2 cytokine levels, IL-4 and IL-5 were 26.2- and 89.3-fold above the background, respectively (Figure 3C and 3D). The CBA results, therefore, confirmed that vaccination of mice orally with the *Salmonella *vaccine vector induced systemic Gag-specific Th1 and Th2 cytokine responses.

### Oral vaccination of mice with recombinant *Salmonella *induces Gag-specific antibodies

**Figure 4:**
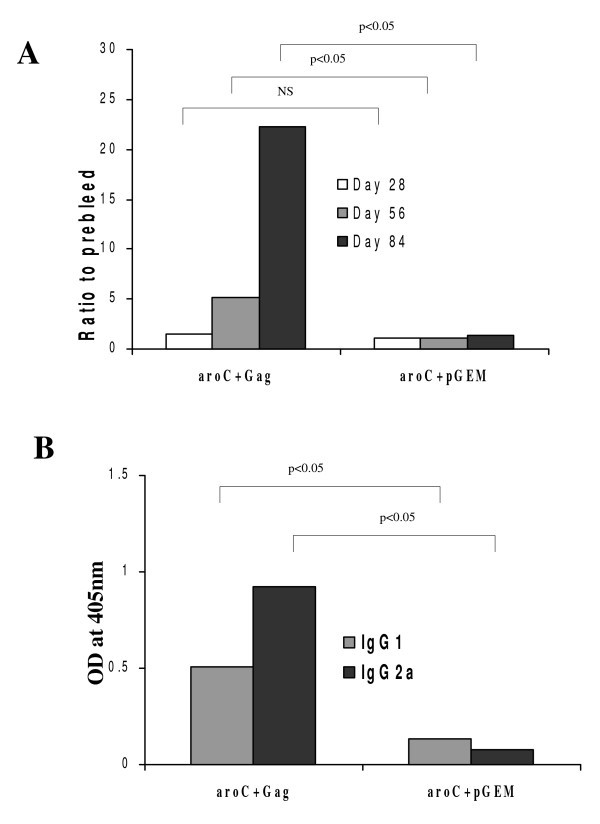
**HIV-1 subtype C Gag-specific serum IgG responses in mice vaccinated with recombinant *Salmonella *vaccine vector**. Groups of mice (5 per group) were vaccinated with live recombinant *Salmonella *vaccine that expressed HIV-1 Subtype C Gag (aroC+Gag) or an antigen-negative *Salmonella *control vaccine (aroC+pGEM) as indicated in Figure 2. Serum (pooled from 5 mice per group) was isolated from blood taken before each vaccination on day 0, 28 and 56 and just before sacrifice on day 84. (A) The HIV-1 Gag-specific IgG for each group of mice with a 1/100 serum dilution. The data are the ratio of the OD_405 nm _for vaccinated mice and the OD_405 nm _for the day 0 serum (pre-bleed). (B) The HIV-1 Gag-specific IgG1 and IgG2a were measured in serum of each group of mice on Day 84 with a 1/100 serum dilution. Each bar represents the mean OD_405 nm _value. Differences in antibody responses between vaccine groups at different time points were analyzed by a two-sample student's t-test and a p < 0.05 was considered statistically significant. A p > 0.05 was considered not statistically significant (NS).

HIV-1 Gag-specific humoral immune responses in mice vaccinated with the HIV Gag-expressing *Salmonella *vaccine were evaluated on days 28, 56 and 84. Anti-Gag total IgG and IgG subtypes IgG2a and IgG1 in the serum of vaccinated mice were determined. A very low serum HIV-1 Gag specific IgG response (1.52-fold OD_405 _reading above prebleed) was detected on day 28 in serum (1/100 dilution) in mice vaccinated with aroC+Gag and this was not significantly above the control (p > 0.05) (Figure 4A). On day 56, the antibody response was boosted significantly (5-fold OD_405 _reading above prebleed) (p < 0.05). The Gag-specific antibody response was further boosted by day 84 (22-fold OD_405 _reading above prebleed) (p < 0.05) (Figure 4A). HIV-1 Gag-specific IgG responses were confirmed using the New LAV Blot I HIV-1 Western blotting kit (Biorad). The serum from the aroC+Gag-vaccinated mice reacted specifically with Gag bands (P55, P40, P24/25 and P17/18) on the blot (results not shown). Gag-specific serum IgG subtypes measured on day 84 indicated the presence of significant (p < 0.01) levels of IgG1 and IgG2a (p < 0.05) (Figure 4B). The Gag-specific Ig2a response was slightly greater than the IgG1 response. In summary, both Th2-related IgG1 and Th1-related IgG2a antibody responses were induced in the mice vaccinated with the HIV Gag-expressing *Salmonella *vaccine.

## Discussion

Delivery of heterologous antigens through the mucosal surface by recombinant *Salmonella *vectors is a powerful strategy for inducing both mucosal and systemic immune responses. After oral vaccination, the *Salmonella *bacteria invade the mucosal surfaces and spread through the mesenteric lymph nodes to distal sites, such as spleen and liver [16,17]. This normally results in the induction of both mucosal and systemic cellular and humoral immune responses [18-20]. In the current study, we investigated the immunogenicity of a recombinant HIV-1 Subtype C Gag-expressing attenuated live *Salmonella enterica *serovarTyphimurium vaccine vector in mice after oral immunization. The HIV-1 *gag *gene was codon-optimized to reflect codons commonly used by *Salmonella *bacteria. The presence of rare codons in foreign genes may affect mRNA and plasmid stability and, in some cases, protein synthesis and bacterial growth [21-24]. In this study, we found that the expression of the HIV-1 Gag in *Salmonella *was improved when the gene was codon-optimized confirming published data [25]. However, other studies have shown that the level of antigen expression was decreased in *Salmonella *when the gene was codon-optimized [26]. Codon-optimization of genes for expression in recombinant *Salmonella *vaccine vectors has been reported to have an impact on the nature, breadth and magnitude of the immune responses induced after vaccination. It has been shown that antigen-specific immune responses against a *Salmonella*-based vaccine that expressed human papillomavirus type 16 L1 improved after codon-optimization [26]. The expression of measles virus (MV) epitopes in a *Salmonella *vaccine vector has been shown to be enhanced by codon-optimization [27]. Oral vaccination of MV-susceptible mice with the recombinant *Salmonella *vector induced MV-specific serum antibodies and CD4+ T cell response [26]. Codon-optimization of HIV-1 *gag *for expression in *Salmonella *resulted in enhanced mucosal immunity in vaccinated mice [25].

Our recombinant HIV-1 Gag-expressing *Salmonella *vaccine induced specific Th1 and Th2 cytokine responses in the spleen. This indicates that the recombinant bacteria successfully delivered the heterologous HIV-1 antigen to the systemic immune system after oral vaccination. Live *Salmonella *can be taken up by antigen-presenting cells by the process of phagocytosis and the bacteria are able to reside and replicate in the phagosomes. The *Salmonella *antigens are, therefore, presented to MHC class II-restricted CD4+ T cells [28-30]. This elicits antigen-specific Th1 and/or Th2 cytokine responses [31-34]. The two types of responses (CD4+ Th1 and Th2) induced by the recombinant *Salmonella *vector in this study are crucial for vaccines that are required to induce both cell-mediated and antibody responses for protection against infection by a number of pathogens. CD4+ Th1 cytokines such as IFN-γ and TNF-α provide protective immunity against intracellular pathogens such as viruses. They promote CD8+ T cell responses and B cell class-switching to IgG2a [35,36]. In contrast, CD4+ Th2 cytokines such as IL-4 and IL-5 promote B cell class switching to neutralizing antibodies such as IgG1 and they further regulate the magnitude of Th1 cytokine responses [36-38]. In this study, we also tested for the induction of Gag-specific CD8+ T cells in vaccinated mice. No specific CD8+ T cells were detectable by ELISPOT and CBA assays (results not shown). This was not unexpected; the secretion of antigens from the *Salmonella *bacteria has been shown to result in induction of antigen-specific CD8+ T cell responses in vaccinated mice [39-41]. Our HIV-1 Gag antigen was expressed inside the bacterial cytoplasm as inclusion bodies and was therefore unlikely to induce potent antigen-specific CD8+ T cell responses.

The nature of immune responses found in this study has relevance to the field of HIV-1 vaccinology. In HIV-1 infection, CD4+ Th1 and Th2 cells play regulatory roles in controlling infection and replication [42,43]. HIV-infected long-term non-progressors have been found to have strong CD4+ T cell responses to HIV-1 antigens such as Gag [44]. Recent studies have also suggested that HIV-specific CD4+ Th1 cell that produce INF-γ and IL-2 are important in long-term reduction of HIV viremia [45,46]. Other studies have shown that the loss of CD8+ T cell responses in HIV-1 infection could be reversed by vaccine-induced CD4+ Th cell responses [47]. Although CD4+ Th cells provide immunological help to CD8+ T cells and B cells, they can also play a more direct role in antiviral activity [48]. CD4+ T cells, like CD8+ T cells, have cytolytic activities against HIV-infected cells and can provide protective immune responses [49-52]. The cytokines, such as IFN-γ and TNF-α, secreted by activated CD4+ T cells have direct antiviral activities [53]. HIV-1 vaccines should, therefore, provoke both specific CD8+ and CD4+ T cell responses, so as to maximize the chance of preventing or controlling infection.

The development of an HIV-1 vaccine that elicits protective humoral immune responses is still a challenge to the scientific community. Such antibody responses should be able to neutralize many strains of the virus if they are to be useful. Although HIV-1 Gag is not a target for neutralizing antibodies, antibodies to Gag may play a role in other responses such as antibody-mediated cellular cytotoxicity and complement-mediated lysis of HIV-1 virions [54]. In the current study, we investigated whether *Salmonella *that express codon-optimized Gag could induce HIV-1 specific antibodies. Gag-specific IgG responses were induced in vaccinated mice. Further characterization of the anti-HIV-1 Gag antibody responses induced after secondary vaccinations with aroC+Gag showed the presence of both IgG1 and IgG2a subclasses. This result was supported by the finding that aroC+Gag induced Gag-specific CD4+ Th1 and Th2 cytokines. These cytokines produced by the Th1 and Th2 cells were most probably responsible for the induction of heavy-chain isotype switching to both IgG2a and IgG1 respectively [36]. In summary, our results highlight the potential of using recombinant *Salmonella *as a vector for HIV-1 antigens. A *Salmonella*-delivered HIV-1 vaccine would be convenient for mass-vaccinations and inexpensive to produce. This would be advantageous for developing countries, where the HIV/AIDS is most prevalent, and the pandemic urgently needs to be brought under control.

## Conclusion

In conclusion, the study showed that oral vaccination of mice with *Salmonella Typhimurium *vector expressing codon-optimized HIV-1 Gag could result in systemic HIV-1-specific CD4+ Th1 and Th2 cell immune responses, together with IgG1 and IgG2a humoral responses. Vaccines that provoke this type of immune response may be important in the prevention or control of HIV-1 infection.

## Abbreviations

CBA: cytometric bead array; ELISA: enzyme-linked immunosorbent assay; ELISPOT: enzyme-linked immunospot; Gag: HIV group antigen; HIV-1: human immunodeficiency virus type 1; IFN-γ: interferon-gamma; IgG: immunoglobulin G; IL-4: interleukin 4; IL-5: interleukin 5; NS: not significant; OD: optical density; SDS-PAGE: sodium dodecyl sulphate-polyacrylamide gel electrophoresis; SFUs: spot-forming units; Th: T-helper; TNF-α: Tumour necrosis factor alpha; 2YT: 2× Yeast Tryptone

## Competing interests

The authors declare that they have no competing interests.

## Authors' contributions

NC, WRB, AW and EGS planned and designed the experiment. NC performed all the experiments. NC, WRB, AW and EGS all participated in the drafting of the manuscript. All the authors read and approved the manuscript.
